# Targeted Proteomics Allows Quantification of Ethylene Receptors and Reveals SlETR3 Accumulation in Never-Ripe Tomatoes

**DOI:** 10.3389/fpls.2019.01054

**Published:** 2019-08-29

**Authors:** Yi Chen, Valérie Rofidal, Sonia Hem, Julie Gil, Joanna Nosarzewska, Nathalie Berger, Vincent Demolombe, Mondher Bouzayen, Beenish J. Azhar, Samina N. Shakeel, G. Eric Schaller, Brad M. Binder, Véronique Santoni, Christian Chervin

**Affiliations:** ^1^GBF, Université de Toulouse, INRA, Toulouse, France; ^2^BPMP, CNRS, INRA, Montpellier SupAgro, Université de Montpellier, Montpellier, France; ^3^Department of Biochemistry, Quaid-i-azam University, Islamabad, Pakistan; ^4^Department of Biological Sciences, Dartmouth College, Hanover, NH, United States; ^5^Department of Biochemistry, Cellular, and Molecular Biology, University of Tennessee, Knoxville, TN, United States

**Keywords:** ethylene, receptor, hormone, signaling, tomato

## Abstract

Ethylene regulates fruit ripening and several plant functions (germination, plant growth, plant-microbe interactions). Protein quantification of ethylene receptors (ETRs) is essential to study their functions, but is impaired by low resolution tools such as antibodies that are mostly nonspecific, or the lack of sensitivity of shotgun proteomic approaches. We developed a targeted proteomic method, to quantify low-abundance proteins such as ETRs, and coupled this to mRNAs analyses, in two tomato lines: Wild Type (WT) and Never-Ripe (NR) which is insensitive to ethylene because of a gain-of-function mutation in ETR3. We obtained mRNA and protein abundance profiles for each ETR over the fruit development period. Despite a limiting number of replicates, we propose Pearson correlations between mRNA and protein profiles as interesting indicators to discriminate the two genotypes: such correlations are mostly positive in the WT and are affected by the NR mutation. The influence of putative post-transcriptional and post-translational changes are discussed. In NR fruits, the observed accumulation of the mutated ETR3 protein between ripening stages (Mature Green and Breaker + 8 days) may be a cause of NR tomatoes to stay orange. The label-free quantitative proteomics analysis of membrane proteins, concomitant to Parallel Reaction Monitoring analysis, may be a resource to study changes over tomato fruit development. These results could lead to studies about ETR subfunctions and interconnections over fruit development. Variations of RNA-protein correlations may open new fields of research in ETR regulation. Finally, similar approaches may be developed to study ETRs in whole plant development and plant-microorganism interactions.

## Introduction

Ethylene is a plant hormone involved in many developmental processes such as seed germination, root initiation, root hair development, flower development, sex determination, fruit ripening, senescence, and responses to biotic and abiotic stresses (Merchante et al., 2013). Recent research has shown that ethylene sensing is also found in cyanobacteria, such *Synechocystis* (Lacey and Binder, 2016) and possibly in early diverging fungi, such as *Rhizophagus* (Hérivaux et al., 2017).

Ethylene gas is perceived by specific receptors (EThylene Receptors, ETRs) localized at the endoplasmic reticulum (Chen et al., 2002). Since the initial description of the first ethylene receptor, AtETR1 from *Arabidopsis thaliana* (Chang et al., 1993), several studies combining genetics, molecular biology, and biochemistry have led to a model whereby the receptors function as negative regulators and ethylene releases this inhibition (Shakeel et al., 2013; Lacey and Binder, 2014; Ju and Chang, 2015). Thus, ETR abundance may be a critical determinant of ethylene signaling. This is supported in tomato where a study showed that the level of insensitivity to ethylene is related to the expression level of an ETR1 gain-of-function (GOF) mutant (Gallie, 2010). Additionally, other authors observed that ethylene insensitivity, due to a receptor GOF mutant, can be partially overcome with increased gene dosage of WT gene (Hall et al., 1999). In other words, the ethylene signaling may be governed by the relative amount of WT ETRs versus mutant ETRs.

A major bottleneck in understanding ETR roles is the absence of a method to quantify the protein levels of all receptor isoforms in the same sample mainly due to the absence of specific antibodies against ETRs (Chen et al., 2002; Kevany et al., 2007; Mata et al., 2018). Hence, two studies correlating receptor protein abundance using antibodies to transcript levels of each ETR isoform made conflicting observations (Kevany et al., 2007; Kamiyoshihara et al., 2012) raising the need for a better method of ETR protein detection. To reach this objective, a targeted mass spectrometry proteomic method, called parallel reaction monitoring (PRM) was recently described to study ETR receptor abundance in tomato fruit (Mata et al., 2018). We adapted this strategy, focusing on single peptides of rare proteins, to compare the abundance of ETRs in WT and in the NR mutant. In this mutant, *ETR3* harbors a Pro36Leu mutation in the ethylene-binding domain, which renders the plant ethylene insensitive to block fruit ripening as well as downregulating the mRNA levels of *ETR1* and *ETR4* at Breaker stage (Hackett et al., 2000). Additionally, these authors showed that the NR fruit changes from green to orange, but never completes ripening by turning red, due to a lack of lycopene accumulation at the end of the ripening period.

## Results and Discussion

### Development of the PRM Analyses for the Seven Tomato ETRs

To better understand the ETRs roles in the control of important traits such as tomato fruit ripening, it is critical to have a method to quantify the levels of all receptor isoforms at different developmental stages. The tomato (*Solanum lycopersicum*) genome encodes seven ETR isoforms (SlETR1 through SlETR7). Recent advances in large-scale shotgun proteomics have led to identify a large set of proteins including SlETR3 and SlETR4 in green to red ripe tomato fruits using the ITAG 2.3 database (Feb 2013) (Szymanski et al., 2017) and SlETR1, 3 and 4, using the UniProt FASTA database (Dec 2015) in red ripe tomatoes (Mata et al., 2017). In a large-scale label-free proteomic study, we identified SlETR1, 4, 6, and 7 using the most recent ITAG 3.2 (June 2017), in pooled skin and flesh tissues of both the WT and NR genotypes of the MicroTom cultivar, in four development stages from immature green to Breaker + 8 days (**Table S2a**; **Methods S1b**). These four ETRs were identified but not quantified in all fruit development stages (**Table S2b**). Such large-scale shotgun studies can identify thousands of proteins in biological samples but may result in an under representation of low-abundance proteins such as ETRs. In contrast, targeted approaches such as the PRM performed on quadrupole-Orbitrap mass spectrometers, offers clear advantage in targeting and quantifying low-abundance analytes (Bourmaud et al., 2016).

**Figure 1:**
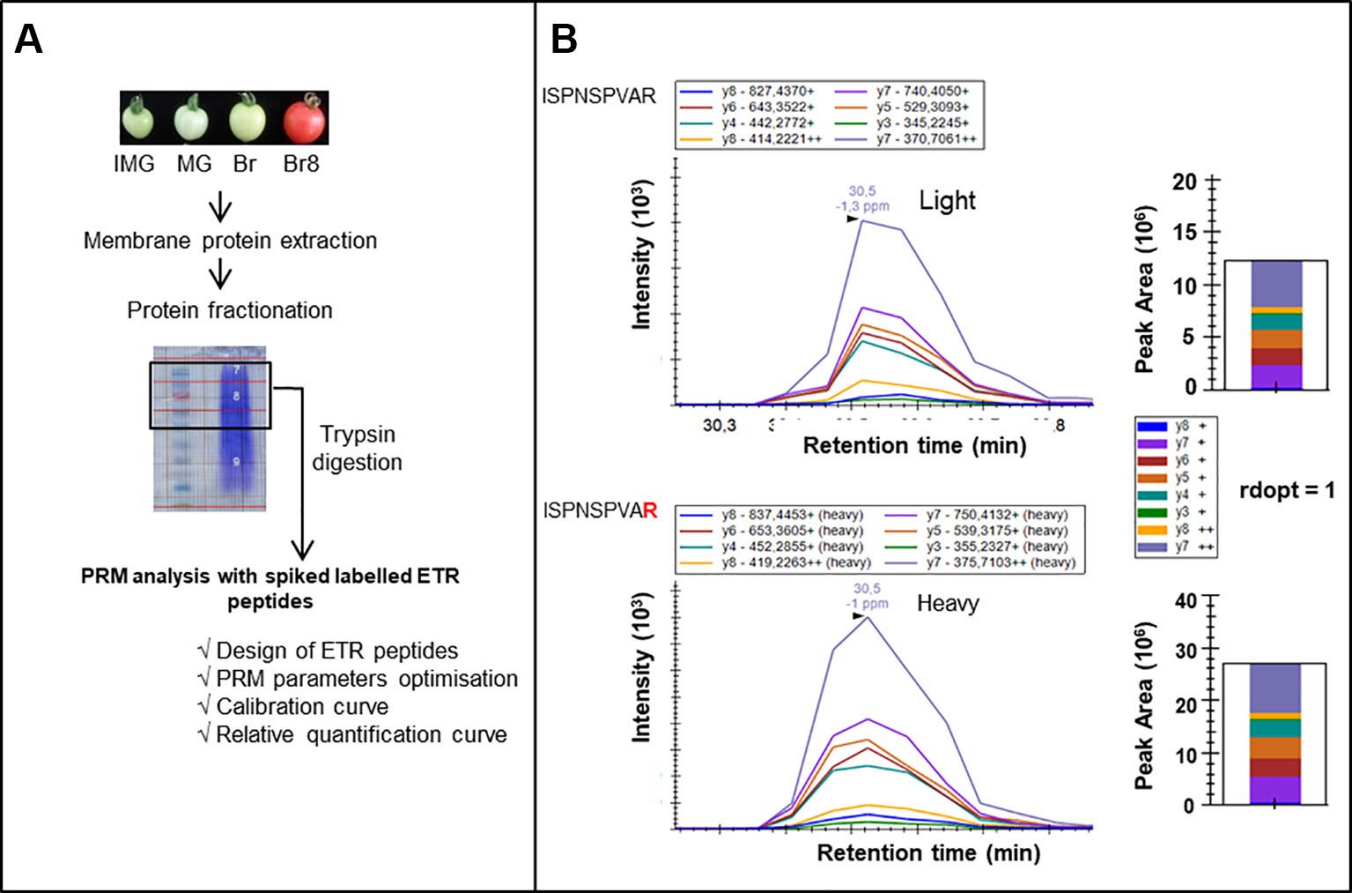
PRM workflow for identification and relative quantification of ethylene receptors. **(A)** Tomato fruits (*Solanum lycopersicum*) from wild-type (WT) plants and NR (never ripe) mutants were collected at four developmental stages: IMG, ImMature Green; MG, Mature Green; Br, Breaker; and Br8, Breaker + 8 days. Membrane proteins were extracted and fractionated through SDS-PAGE electrophoresis. Proteins above 50 kDa were digested with trypsin, and peptides were injected in LC-MS/MS [(nano-HPLC coupled to a quadrupole Orbitrap Qexactive + (Thermo)]. A PRM analysis was optimized through i) the design of ETR peptides, ii) the optimization of PRM parameters, and iii) calibration curves with labeled peptides to identify and relatively quantify ETRs. **(B)** LC−PRM data validating the identification of ETR1. Heavy peptide (ISPNSPVAR) was spiked into IMG WT biological sample. Selected transitions were extracted for the heavy and endogenous peptides, and rdopt value was calculated using Skyline software (see *Materials and Methods*).

A PRM strategy was thus developed to identify ETRs in tomato fruit over the ripening period (**Figure 1A**). Microsomal proteins were extracted from tomato fruits at four developmental stages. Proteins were fractionated through SDS-PAGE gel electrophoresis and subsequently digested by trypsin (**Figure 1A**). The success of a PRM-based targeted assay depends on choosing the most appropriate proteotypic peptides for use as specific tracers of each of the proteins of interest (Bourmaud et al., 2016). An *in silico* analysis was performed in order to discriminate between the 7 ETRs and 16 labeled proteotypic peptides (at least 2 proteotypic peptides/ETR) were synthesized (**Table S1a**) and used in a PRM approach to identify the corresponding endogenous ETRs (**Figure 1B**, **Figure S1**).

**Figure 2:**
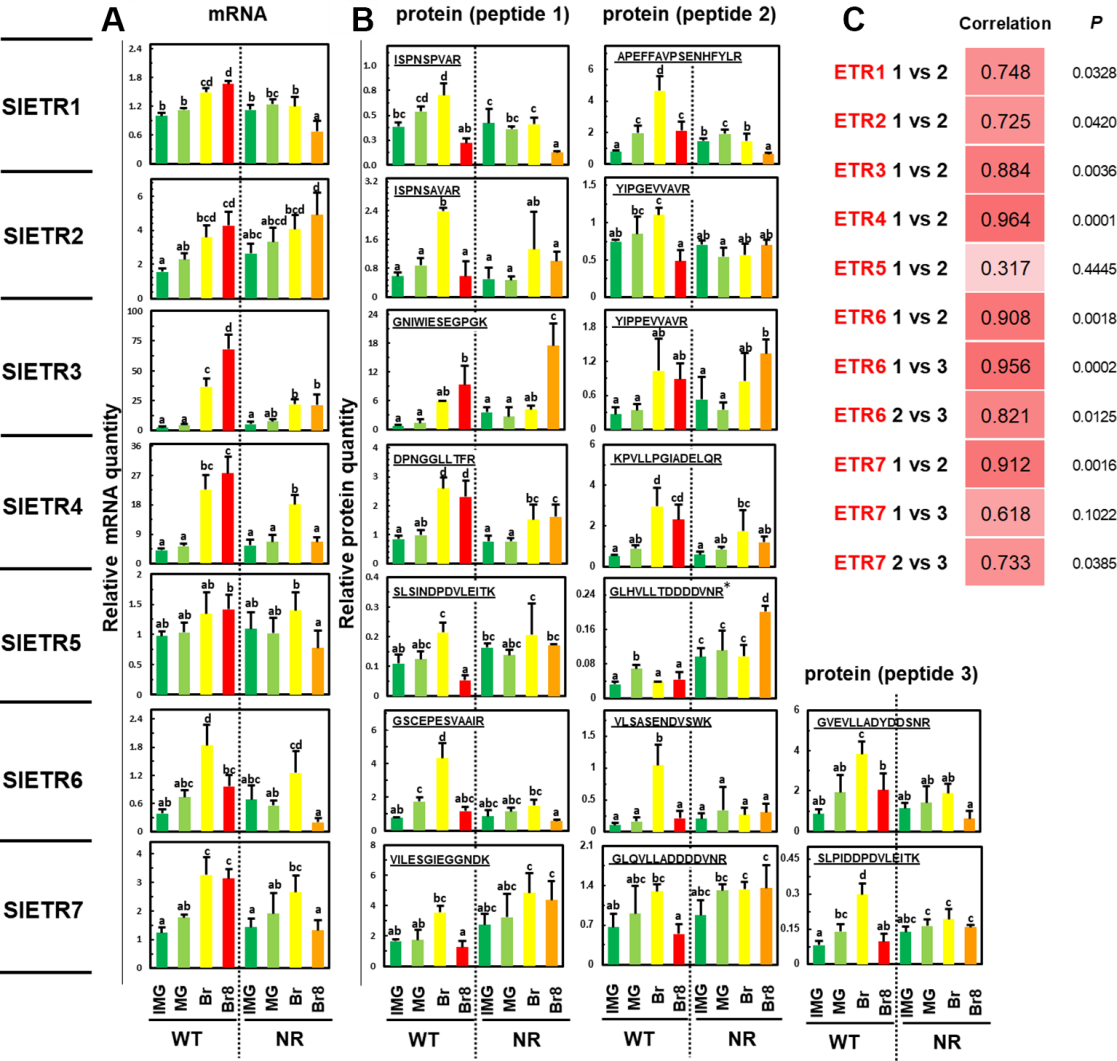
Variations of abundance of mRNAs and proteins of the seven ETRs over fruit development in WT and NR genetic variants of *Solanum lycopersium*, cv. MicroTom. Four development stages were sampled: immature green (IMG, dark green bars), mature green (MG, light green bars), breaker (Br, yellow bars), and breaker + 8 days (Br8, red bars in WT, orange bars in NR). The results show **(A)** the quantities of RNAs relative to that of ETR1 at the IMG stage and **(B)** the relative quantity of protein based on the ratio between endogenous and spiked labeled peptide (see *Materials and Methods* for calculation details). See **Table S1** for details about peptides “1, 2, and 3” for each ETR. The results are the means of three independent biological replicates, error bars show SE, and the small letters show significant differences at 0.05 level (Fisher’s LSD test). *Peptide at the bottom limit of the linear regression in dynamic range. **(C)** Pearson correlation coefficients between the profiles of the various specific peptides tested in this study. *P* is the probability of the correlation; the power values were calculated at the 0.05 risk.

Among the 16 proteotypic ETR peptides, 15 were identified with high confidence (rdopt > 0.95), except the peptide GLHVLLTDDDDVNR that belongs to ETR5 (rdopt = 0.94) (**Table S1a**, **Figure S1**). Thus, the seven ETRs encoded by the tomato genome were identified in the two genotypes whatever the developmental stage (**Figure 2B**, **Figure S1**). To quantify the ETRs over fruit maturation, the labeled peptides were spiked into a biological matrix using seven adapted peptide concentrations to obtain calibration curves used to determine their quantification limit (**Figure S2**). All identified peptides showed linear regressions with regression coefficients above 0.90 allowing their relative quantification (**Figure S2**). The accumulation profiles of the different peptides for each ETR revealed high correlation coefficients (**Figure 2C**) except in the case of ETR5, likely due to a low protein accumulation during fruit ripening and a limited dynamic range (**Figure S2**). However, the power that reflects the reproducibility of the significance (Zhang and Wen, 2019) appears low with either ETR1 or ETR2 or ETR7 pep3, suggesting that more replicates would be necessary to make better predictions.

### Changes in the Seven ETR Proteins Over Tomato Development in WT and NR Backgrounds

Using PRM, we successfully measured the relative amount of the seven ETRs in a series of ripening tomatoes (**Figure 2B**). This showed that the protein levels of ETR1, 2, 5, 6, and 7 dropped from Br to Br8 stages in WT, but this was not the case with ETR3 and ETR4 (**Figure 2B**) indicating that there is a differential regulation of ETRs. In addition, one interesting result is an accumulation of ETR3 in the NR fruit between the mature green stage (MG), and the Breaker + 8 days stage (Br8), which delimits the ripening phase (**Figure 2B**) (Hoeberichts et al., 2002). ETR3 is mutated in the NR background rendering the plant insensitive to ethylene (Wilkinson et al., 1995), and tomato fruit ripening has previously been shown to be blocked by GOF mutations in ETR1 (Okabe et al., 2012). However, since protein content was not determined in earlier studies (Wilkinson et al., 1995; Okabe et al., 2012), our study brings further understanding on how ripening may be blocked in NR fruits at the ETR protein level. Various studies indicate that ethylene acts as a negative regulator. In this model, in air without ethylene, the receptors output leads to inhibition of the ethylene signaling pathway. When ethylene is present, it alleviates this inhibition (Shakeel et al., 2013). Receptors that cannot bind ethylene, such as the mutant ETR3 receptor in the NR background, are thus incapable of turning off. Based on this, we propose that the low levels of the mutant ETR3 in the NR at the early stages of fruit ripening only leads to partial ethylene insensitivity because there is not enough mutant receptor to mask ethylene perception when the other receptor isoforms bind ethylene. In contrast, when mutant ETR3 levels increase at later stages during ripening, the increased signaling from the mutant receptor masks the perception of ethylene by the other receptors.

**Figure 3:**
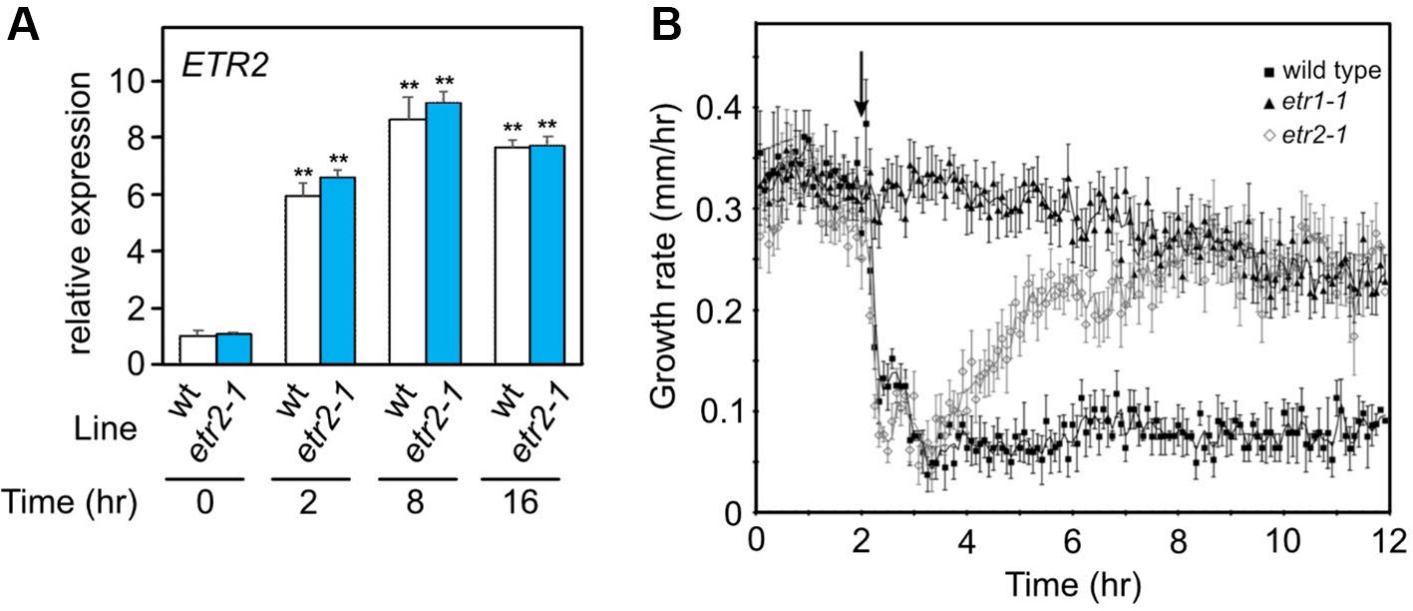
**(A)** Gene expression based on qPCR for *ETR2* alleles of dark-grown WT and etr2-1 seedlings treated for the indicated times with 10 µL L^−1^ ethylene. Expression was normalized to a tubulin control and is presented as relative to the untreated WT control. Statistical analysis was performed using one-way ANOVA with *post hoc* Tukey HSD test for comparison of induction compared to the 0 time point for each genotype (** *P* < 0.01); n = 3 biological replicates. No significant difference was found between the *ETR2* allele expression levels in WT and etr2-1 at any time point (t-test, *P* > 0.05). Error bars show SE. **(B)** Effect of *etr1-1* and *etr2-1* on the short-term ethylene response in etiolated *Arabidopsis* seedlings. A kinetic analysis of hypocotyl growth was carried out on *etr1-1* and *etr2-1* mutants, by time lapse imaging. For comparison, Columbia (wt) seedling responses are included. The seedlings were grown in air for 2 h, at which time, 10 µL L^−1^ ethylene was added. The average ± SEM from at least six seedlings is shown.

To evaluate whether such dynamic regulation is possible, we examined the ethylene growth inhibition kinetics of hypocotyls of two *Arabidopsis* ethylene receptor mutants, *etr1-1* and *etr2-1*. The *etr1-1* plants are ethylene insensitive, and *etr2-1* has a large reduction in ethylene sensitivity (Bleecker et al., 1988; Chang et al., 1993; Sakai et al., 1998). *ETR1* is constitutively expressed, whereas *ETR2* occurs at low levels in air and is induced by ethylene within 2 h (Binder et al., 2004a; Hua and Meyerowitz, 1998). Similarly, we observed an induction of *etr2-1* by ethylene (**Figure 3A**). We predicted that if this model of regulation is correct, *etr1-1* seedlings should show no response to ethylene. In contrast, the *etr2-1* seedlings should have a transient growth inhibition response because initially the levels of *etr2-1* are predicted to be too low to block ethylene perception, but upon induction by ethylene, the higher *etr2-1* levels should block ethylene signaling. As shown in **Figure 3B**, WT seedlings had ethylene response kinetics similar to previous studies where growth was inhibited for as long as ethylene was present (Binder et al., 2004b; Binder et al., 2006). In contrast, the *etr1-1* seedlings had no measurable response to ethylene, but did have a slow decline in growth rate over time similar to what has been observed in WT seedlings in air (Binder et al., 2006). Interestingly, *etr2-1* seedlings responded transiently to the application of ethylene with an acceleration in growth rate starting at approximately 2 h after the initial application of ethylene. These results are consistent with our model that proposes that increased levels of a mutant ethylene receptor can cause ethylene insensitivity *in planta*.

The receptors form higher-order complexes much like bacterial chemoreceptors (Shakeel et al., 2013). Thus, in this model, it is possible that the increased levels of mutant receptors are blocking perception by direct interactions between mutant and non-mutant receptors. Alternatively, the increase in mutant receptor levels might be blocking access of WT receptors to downstream effectors such as CTR1. In either case, this model explains why NR fruits start to ripen, but then stop at later stages. This model is consistent with observations in *Arabidopsis* where the ethylene insensitivity of several receptor gain-of-function mutants are overcome by increasing levels of WT receptors (Hall et al., 1999).

**Figure 4:**
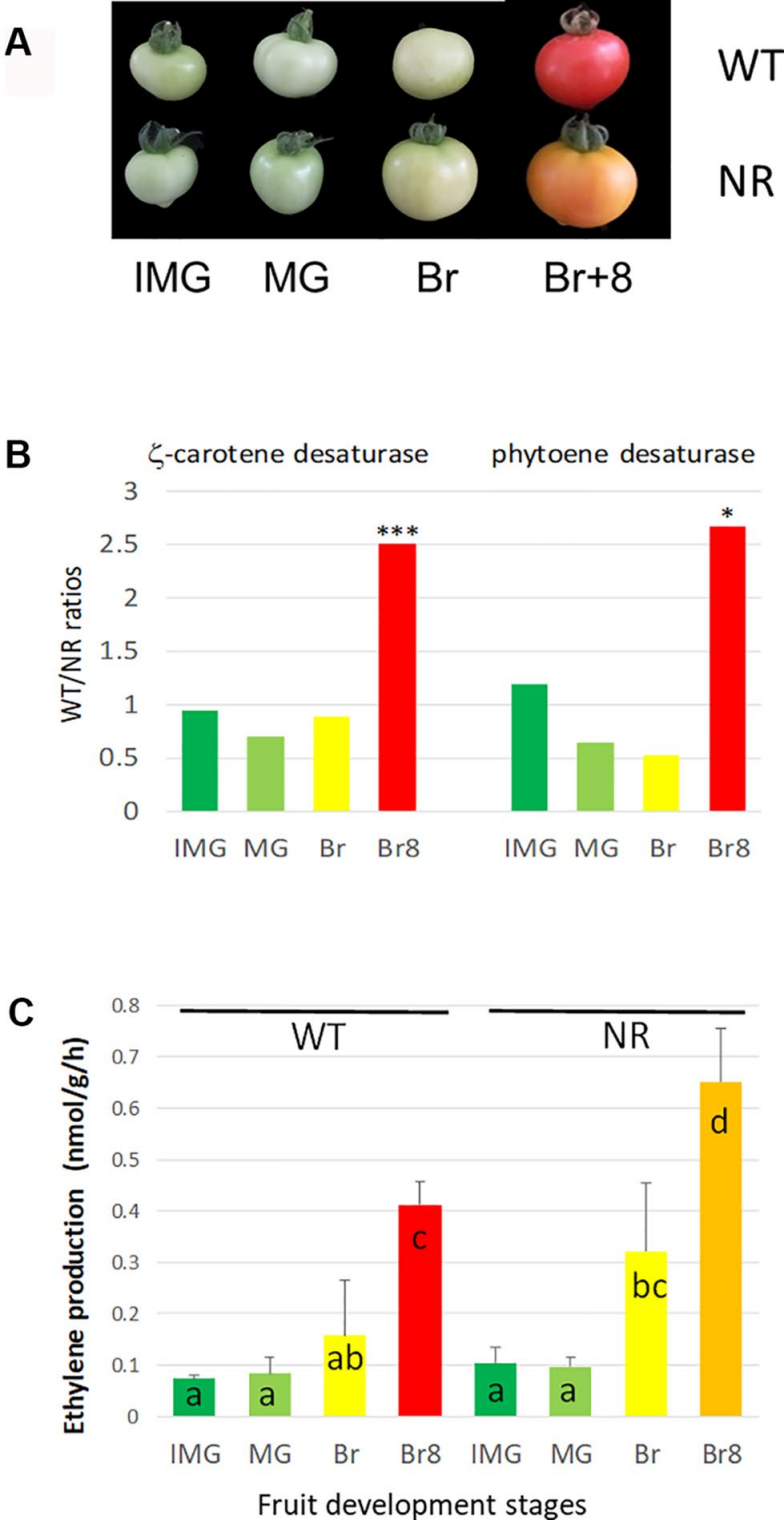
Phenotypes and biochemical changes in WT and NR tomato cultivars. **(A)** Four development fruit stages, used in this study, in two MicroTom tomato lines, WT stands for wild type, and NR stands for never ripe. IMG stands for IMmature Green, MG stands for Mature Green, Br stands for Breaker, and Br + 8 stands for Breaker + 8 days. Both cultivars originate from LE Pereira Peres’ Laboratory (Universidade de São Paulo, Brazil) and have been described previously (see *Materials and Methods*). **(B)** Ratios WT/NR of two enzymes involved in lycopene accumulation as a function of fruit development stages. Data obtained by label-free analysis on the same extracts as for PRM. The protein analysis was performed as described in **Methods S1b**, using the ITAG 3.2 annotation. The ratios were performed by means of three biological replicates (data available in **Supp. Table 2b**). * and *** stands for P < 0.05 and P < 0.001, respectively, resulting of t-test comparisons between WT and NR means at a similar stage. Carotene desaturase: Solyc01g097810, Phytoene desaturase: Solyc03g123760. **(C)** Ethylene production by developing fruits (two lines and four stages as described above). The results are the means of three independent biological replicates, error bars show SE, and the small letters show significant differences at 0.05 level (Fisher’s LSD test).

The NR mutant fruit fails to turn red (**Figure 4A**), and this is due to a limited accumulation of lycopene (red pigment) as previously shown (Liu et al., 2012). Support for this is that using a large-scale label-free quantitative proteomic approach on the same microsomal extracts, with three biological replicates (see **Table S2**), we observed a decreased accumulation of two key enzymes for lycopene synthesis, zeta-carotene desaturase and phytoene desaturase, in the NR samples compared to WT (**Figure 4B**); ratios around 2.5 show enzymes that were 2.5-fold more present in WT than in NR. Lower accumulation of lycopene in fruits has also been observed with a GOF mutation in ETR1, but the abundance of receptor protein was not determined (Okabe et al., 2012). The label-free data available through the ProteomeXchange database are interesting resources to mine for additional changes occurring at the membrane in the tomato fruit development.

Additionally, the NR mutation led to higher C_2_H_4_ production than in WT in ripening tomatoes (**Figure 4C**), which was already observed in ETR1 GOF mutant tomato (Mubarok et al., 2015). Finally, the NR mutation also resulted in higher levels of ETR5 and ETR7 at Br8 (**Figure 2B**). However, because these ETRs are WT proteins and do not harbor mutations altering their sensitivity to ethylene, their higher accumulation is predicted to cause a milder change in ethylene sensitivity, as observed by Hall et al. (1999), as opposed to accumulation of NR, which leads to ethylene insensitivity. Moreover, care should be taken as we observed relative protein values, as discussed below.

Indeed, additional experiments will be necessary to switch from peptide quantification to protein quantification, mainly because of post-translational modifications that can alter the true protein quantification. For instance, the increased abundance of the peptide GNIWIESEGPGK in NR at Br8 stage could be the consequence of *in vivo* serine dephosphorylation, inducing an apparent increase in the quantity of the non-modified peptide. This latter hypothesis would suggest that ETR3 is phosphorylated at this site and less phosphorylated in NR than in WT. It remains an open question whether or not this site is phosphorylated. Other reasons for such discrepancies between peptides are different digestion efficiencies along the protein sequence and partial adsorption of labeled peptides into vials. We obtained similar accumulation profiles for ETR3, ETR4, ETR6, and ETR7 to those reported by Mata et al. (2018) in the WT plant and small differences for ETR1, 2 and 5. Mata et al. (2018) did not test NR. However, there are important differences to note between our study and Mata’s study as they used a different cultivar, very different growth conditions, and different methods for protein extraction. Despite these differences, we think both data sets will shed new light on ethylene signaling during fruit development.

### Are There Positive or Negative Correlations Between the Seven ETR mRNAs and Protein Levels?

Another critical question for ETRs is to understand the relationship between the abundance of mRNA and of the corresponding proteins because prior studies revealed conflicting results about such correlations in the tomato fruit (Kevany et al., 2007; Kamiyoshihara et al., 2012). Therefore, we examined the transcript levels of each *ETR* using qRT-PCR and correlated this information with protein quantification results (**Table S3**, and **Figures 2A, B**). With only four points per correlation, the powers, which represent the reproducibility of the significance, are too weak to make any solid conclusion (**Table S3**). Mata et al. (2018) found positive correlations between RNAs and proteins, but the correlation was only significant for ETR3. In WT, a positive correlation was generally observed between RNA and protein levels (**Figures 2A, B**), and this can be verified by averaging results of pep1 and pep2, then piling up all RNA data and all protein data to generate the Pearson correlation coefficient, with a total of 28 values. The Pearson correlation is then 0.754, the *P* value is 3.5810^−6^, and the power is 0.998. This is very global, and suppresses all possible analyses between the different stages and different ETRs, but at least, it validates the positive correlation proposed by Kamiyoshihara et al., (2012) and invalidates the negative correlation proposed by Kevany et al. (2007). In NR, when comparing RNA and protein levels globally, as described above, the Pearson correlation coefficient with a total of 28 values is then 0.586, the *P* value is 1.0410^−3^, and the power is 0.919. Thus, NR modifies the correlation compared to WT. When comparing WT to NR, at the Br8 stage, the NR mutation caused a decreased accumulation of mRNA of *ETR1*, *ETR3*, *ETR4*, *ETR6*, and *ETR7* (**Figures 2A, B**) suggesting some as yet unknown transcriptional controls. In *Arabidopsis*, the etr1-1 gain-of-function mutation did not cause changes in the transcript levels of the other four receptor isoforms (O’Malley et al., 2005). However, this mutation did result in higher levels of mutant etr1-1 protein compared to ETR1 levels in wild-type plants, even though transcript levels for this receptor were unchanged (Zhao et al., 2002). The mRNA variations observed of the seven ETRs matched previous observations analyzed from various RNAseq in tomatoes (Chen et al., 2018).

### The NR Mutation seems to Affect the Correlation Between mRNA and Protein Levels

The NR mutation also causes several changes in the correlations between mRNA and protein abundance (**Figure 2B**), in particular, in the case of ETR1 where the Pearson coefficient changed from negative in WT (−0.17 and 0.59 for PEP1 and PEP2, respectively) to positive in NR (0.93 and 0.96 for PEP1 and PEP2, respectively) (**Table S3**). However, the weakness of power values would require a higher number of points to strengthen the correlation analysis; thus, **Table S3** is used to give trends at a glance. For the ETR4 and ETR7 receptors, mRNA levels decreased at BR8 in NR with either little to no change in protein levels (**Figures 2A, B**) suggesting that breakdown of these receptors is reduced in the NR mutant background. Further analysis will be required to determine the mechanism by which this occurs. For other ETRs such as ETR4 and ETR6, mRNA/protein correlation coefficients were very high and minimally affected by the NR mutation (**Table S3**); however, the power values were still too low for validating the trends.

## Conclusions

We developed a PRM strategy that allowed the comparison of the abundance of ETRs in NR and WT tomato plants. Because ethylene has important roles in regulating plant development and responses to stresses, this method will be of wide use to study the roles of this phytohormone in diverse responses and plant species. However, calibration will be necessary for each peptide in each plant species. The observation that the GOF mutant ETR3 protein accumulates in orange mature fruit of the NR mutant is an example of regulation that would have remained unknown without the development of this new method. mRNA/protein correlations could also bring information about the regulation that occurs in ethylene signaling in fruit tissues, but more replicates are necessary. Given that ETRs in *Arabidopsis* show patterns of subfunctionalization (Shakeel et al., 2013), the use of PRM in tomato and other plant species will provide critical information about ETR subfunctionalization across the plant kingdom.

## Materials and Methods

### Plant Materials and Growth Conditions

Two tomato lines (*Solanum lycopersicum*) cv. Micro-tom were used, WT and NR mutant (Pro36Leu), both previously described (Carvalho et al., 2011). In addition to fruit color difference, these authors showed that NR seedlings are less sensitive to exogenous ethylene than WT seedlings, a classical response of ETR GOF mutants. Plants were grown in culture rooms with the following conditions: day/night (26°C for 18 h, 18°C for 8 h), light intensity 250 µmol.m^−2^.s^−1^, relative humidity at 80%. Four fruit stages were studied: IMmature Green (IMG), Mature Green (MG) fruit were harvested 20 and 38 days after flower anthesis, respectively; Breaker (Br) fruit was harvested once fruit color changed from green to yellow and red fruit (Br + 8) was harvested 8 days later (**Figure 4A**). Ethylene was analyzed using gas chromatography as previously described (Trapet et al., 2016) by incubating the fruit for 3 h. *Arabidopsis* lines (*Arabidopsis thaliana*) cv. Colombia were used, WT and *etr1-1 and etr2-1*, using growing conditions and growth monitoring described previously (Shakeel et al., 2013, and refs herein).

### mRNA Purification and qPCR Analysis

For each fruit stage, the skin, together with pericarp tissues were collected and divided in three biological replicates of five fruits each, originated from different fruits, then ground to a fine powder in liquid nitrogen using a ball grinder. Total RNAs were purified from 100 mg of frozen sample with ReliaPrep^TM^ RNA Tissue Miniprep System (Promega), according to the manufacturer’s instructions. RNA was treated with DNase I (Invitrogen-AM1906), then 2 μg of RNA was treated with GoScript Reverse Transcriptase (Promega-A5003). Quantitative real-time PCR (qPCR) reactions were performed using 5 ng of cDNA per well as described before (Chervin and Deluc, 2010). EF1α, GAPDH, and actin were selected as house-keeping genes. All primers (**Table S1c**) were designed with primer-blast (https://www.ncbi.nlm.nih.gov/tools/primer-blast/).SlETR1 expression in WT at IMG stage was used as control for all genes at all stages.

### Microsomal Protein Extraction

The fruit samples used for mRNA extraction were also used for protein extraction, performed at 4°C according to previous studies (Bono et al., 1995; Kamiyoshihara et al., 2012) with some modifications. Briefly, 3 g of frozen ground powder was mixed with 25 ml of extraction buffer (50 mM Tris-HCl pH 7.0, 10 mM EDTA, 0.5 M sucrose, 3% PVPP w/v, 10 mM DTT, 100 μM PMSF, cOmplete™ Protease Inhibitor Cocktail (one tablet/100 ml), 1 mM phenantrolin, 1 mM Na-orthovanadate). The slurry was filtered through glass cotton at 300*g*, for 5 min and 900*g* for10 min). Then, left-over tissue bits were removed at 3,000*g* for 15 min. The resulting supernatant was centrifuged at 48,000*g* for 60 min. The pellet was resuspended in 25 mM TrisHCl buffer pH 7.0, 250 mM sucrose, 1 mM CaCl_2_, 1 mM MgCl_2_, and cOmplete^TM^ Protease Inhibitor Cocktail (one tablet/10 ml). Proteins were quantified with DC^TM^ Protein Assay (Bio-Rad). Proteins (80 µg/lane) were fractionated using 10% precast SDS-PAGE gel electrophoresis (Biorad) after incubation at 37°C for 30 min in a loading buffer (50 mM Tris-HCl pH7.0, 10% glycerol, 4% SDS, 100 mM DTT, stained with bromophenol blue). The gels were then stained with Coomassie blue (R250, BioRad), then rinsed with acetic acid/methanol (Destain, BioRad). Each lane was cut in two bands, and bands containing proteins with a molecular weight above 50 kDa (with ETR dimers and monomers) were further analyzed by mass spectrometry.

### Targeted LC-Parallel Reaction Monitoring Analyses

*Protein digestion:* Gel band treatments and trypsin digestion were performed as described in **Methods S1b**. Briefly, proteins in gel slices were reduced, alkylated, and digested overnight at 37°C with modified trypsin at a 1:100 enzyme/protein ratio (Promega, Madison, WI, USA). Peptides were extracted twice by the addition of 200 µL of 80% acetonitrile (ACN) and 2% formic acid (FA), and then dried in a vacuum centrifuge. Peptides were then resuspended in 20 µl FA 2%.

*ETR peptide selection:* To select ETR peptides to be studied in the PRM experiment, ETRs were digested *in silico* using MS digest (ProteinProspector tool, v. 5.19.1, University of California). Search criteria included digestion by trypsin, peptide mass from 500 to 4,000 Da, a minimum peptide length of six amino acids, and a uniqueness in the ITAG 3.2 database digested *in silico*. The peptides should also contain a minimal number of methionine residues because of their putative oxidation, of asparagine and glutamine residues because of their putative deamidation, of glutamic acid or glutamine as first amino acid because of the pyro-glutamination, of serine, threonine, or tyrosine residues because they can be phosphorylated. Then, the presence of proline was privileged because of its property to facilitate the MS/MS fragmentation. In addition, the proteotypic peptides previously identified in shotgun analyses (Mata et al., 2017; Szymanski et al., 2017) were preferentially selected. For the seven selected ETRs, 16 proteotypic peptides were selected. Labeled (or heavy) crude synthetic peptides were synthetized (PEPotec, ThermoFisher Scientific) with carbamidomethylation of cysteins and isotopic labeling of the last sequence amino acid (R: +10 Da (^13^C6, ^15^N4) or K: +8 Da (^13^C6, ^15^N2) (**Table S1a**).

*Parallel Reaction Monitoring (PRM):* Labeled peptides were mixed together in a hand-adjusted concentration-balanced mixture to equilibrate individual peptides signals and spiked in a biological matrix made of IMG WT sample in a similar quantity to the one used in all samples further analysed (**Figure 2** and **Figure S1**). The peptide mixture was analyzed using an UltiMate^TM^ NCS-3500RS Ultra High Performance Liquid Chromatography system interfaced online with a nano easy ion source and a Q Exactive Plus Orbitrap mass spectrometer (ThermoFisher Scientific Inc, Waltham, MA, USA). Peptides were first loaded onto a pre-column (Thermo Scientific PepMap 100 C18, 5 μm particle size, 100 Å pore size, 300 μm i.d. x 5 mm length) from the Ultimate 3000 autosampler with 0.05% trifluoroacetic acid for 3 min at a flow rate of 10 μL/min. Then, the column valve was switched to allow elution of peptides from the pre-column onto the analytical column (Thermo Fisher Scientific Inc, Waltham, MA, USA, C18, 2 μm particle size, 100 Å pore size, 75 μm i.d. x 50 cm length). Loading buffer (solvent A) was 0.1% formic acid (FA) and elution buffer (solvent B) was 80% ACN + 0.1% FA. The three step gradients were 4–25% of solvent B for 103 min, then 25–40% of solvent B up to 123 min, and 40–90% of solvent B from 123 to 125 min, at a flow rate of 300 nL/min. The total chromatographic run time was 150 min including a high organic wash and re-equilibration steps. Peptides were transferred to the gaseous phase with positive ion electrospray ionization at 1.7 kV. Labeled peptides were checked by High-energy Collisional Dissociation MS/MS with regard to their retention time, charge, and m/z (**Table S1a**). A schedule PRM method was developed to simultaneously target all peptides (16 light peptides and 16 heavy peptides) in the protein sample (analytical details provided in **Methods S1a**). The Q-Exactive Plus Orbitrap instrument was operated as follows: a full MS scan spectra considering a mass range of 350–2,000 m/z was acquired with a resolution of 17.500 with an automatic gain control (AGC) fixed at 3e^6^ ions and a maximum injection time set at 100 ms. Targeted MS/MS spectra were acquired with a resolution of 140.000 with an AGC fixed at 2e^5^ and with the maximum injection time set at 1,000 ms. An MS/MS spectral library was acquired using a mixture of 16 heavy labeled synthetic peptides (**Methods S1a**). After manual checking of effective co-elution of endogenous and isotopically labeled peptides and after elimination of transitions showing interference, the Rdot-product (rdotp) values were calculated with Skyline (MacLean et al., 2010) (**Figure S1**), and peptides were relatively quantified with at least four transitions (**Figure 1**, **Table S1a**, and **Figure S1**).

Calibration curve was established using stable isotope-labeled peptides spiked into WT IMG samples prior to LC–MS/MS analysis using seven different peptide concentrations adapted for each peptide (**Figure S2**). Provided that the regression coefficient was above 0.90 and the rdopt was above 0.95, the peptide was qualified to be further quantified. For each peptide, the ratios of the endogenous to labeled peak areas were compared to obtain a relative quantification according to the genotypes and the development stages, as follows: relative level of endogenous peptide = sum of all transition intensities of the endogenous/sum of all transition intensities of the labeled.

The Pearson correlations have been calculated using R code, *via* the Wessa online tool (https://www.wessa.net/rwasp_correlation.wasp). The powers of the Pearson correlations were calculated using Sigmaplot (Systat Sotware, Inc.) at the 0.05 level.

### Quantitative Real-Time PCR

For qPCR of *Arabidopsis* seedlings, 4-day-old dark-grown seedlings were grown in hydrocarbon-free air as described (Hall et al., 2012) and treated with 10 µL of L-1 ethylene for the indicated times at the end of their growth cycle. Total RNA was extracted from seedlings using the E.Z.N.A. Plant RNA Kit (Omega Bio-Tek), DNase treatment was performed using TURBO DNA free kit (Invitrogen), and cDNA was synthesized using the SuperScript III First Strand cDNA Synthesis Kit (Invitrogen, USA) according to the manufacturer’s instructions. Real-time PCR was performed using iTaq Universal SYBR Green Supermix (Bio-Rad) and primer sets specific for ETR2 (5′-AGAGAAACTCGGGTGCGATGT-3′ and 5′-TCACTGTCGTCGCCACCATC-3′) and b-tubulin (At5g62700) control (5′-TGGTGGAGCCTTACAACGCTACTT-3′ and 5′-TTCACAGCAAGCTTACGGAGGTCA-3t).

### Time Lapse Imaging

Ethylene growth response kinetics of etiolated *Arabidopsis* seedlings were determined according to methods previously described (Binder et al., 2004a and Binder et al., 2004b) on 2-day-old, dark-grown *Arabidopsis* seedlings grown on 0.8% (w/v) agar plates with half-strength Murashige and Skoog medium at pH 5.7 (Murashige and Skoog, 1962).

## Data Availability

The PRM data are deposited to PeptideAtlas, accessible *via* ftp://PASS01274:DB4724xpa@ftp.peptideatlas.org/, Username: PASS01274, Password: DB4724xpa; and the label-free data are deposited to ProteomeXchange with the dataset identifier PXD011412, Username: reviewer72717@ebi.ac.uk, Password: g7EGcQI4.

## Author Contributions

CC and VS conceived the study. YC performed tomato culture, fruit sampling, protein extraction and purification, and mRNA extraction and analyses. VR performed protein digestion and MS/MS analyses. VR and SH designed specific peptides with help by JG, JN, and NB, who performed preliminary studies. VR, SH, and VD analyzed the MS/MS data. BA performed Arabidopsis gene expression analysis under the supervision of SS and GS. BB performed Arabidopsis kinetic analysis. BA, SS, BB, and GS analyzed and interpreted Arabidopsis data. YC, BB, VR, SH, MB, VS, and CC interpreted the data and wrote the manuscript. All authors read and approved the final manuscript.

## Funding

The authors acknowledge CSC for the PhD studentship to Y Chen, and INRA for a Starter Research grant to V Santoni and C Chervin. Part of this work was supported by the National Science Foundation Grants IOS-1456487 to GS and MCB-1517032 to GS and BB, and the International Research Support Initiative Program of Higher Education Commission of Pakistan to BA. 

## Conflict of Interest Statement

The authors declare that the research was conducted in the absence of any commercial or financial relationships that could be construed as a potential conflict of interest.

The handling editor is currently organizing a Research Topic with one of the authors MB, and confirms the absence of any other collaboration at the time of review
