# Supplementary material for: Targeted Proteomics Allows Quantification of Ethylene Receptors and Reveals SlETR3 Accumulation in Never-Ripe Tomatoes
Source: Front Plant Sci. 2019 Aug 29;10:1054. doi: 10.3389/fpls.2019.01054 (PMC6727826; doi:10.3389/fpls.2019.01054)
Supplement: Supplementary file 1 [file DataSheet_1.zip › 1234Table S3 Correlation ETR RNAs proteins.docx]

**Supporting Table S3:** Pearson correlation coefficients between RNA quantity and peptide quantity of Figures 2a and 2b. The coefficients indicate a positive or negative correlation, and the strength of the correlation (high when close to 1 or -1). PEP1, PEP2 and PEP3 stand for peptide numbers as they appear in Fig. 2 and in Supp Table S1b. *P* is the probability of the correlation, low when there is an association between the variables; the power values were calculated at the 0.05 risk.

|  | ETR1 | | ETR2 | | ETR3 | | ETR4 | |
| --- | --- | --- | --- | --- | --- | --- | --- | --- |
|  | RNA/PEP1 | RNA/PEP2 | RNA/PEP1 | RNA/PEP2 | RNA/PEP1 | RNA/PEP2 | RNA/PEP1 | RNA/PEP2 |
| Correl WT | -0.17 | 0.59 | 0.32 | -0.19 | 0.99 | 0.84 | 0.95 | 0.92 |
| Correl NR | 0.93 | 0.96 | 0.72 | 0.05 | 0.60 | 0.83 | 0.53 | 0.90 |
| *P* WT  Power WT | *0.835*  0.037 | *0.41*  0.100 | *0.68*  0.052 | *0.81*  0.039 | *0.00*  0.967 | *0.16*  0.230 | *0.05*  0.449 | *0.08*  0.355 |
| *P* NR  Power NR | *0.07*  0.381 | *0.04*  0.494 | *0.28*  0.146 | *0.95*  0.028 | *0.40*  0.103 | *0.17*  0.220 | *0.47*  0.085 | *0.10*  0.313 |
|  |  |  |  |  |  |  |  |  |
|  | ETR5 | | ETR6 | | | ETR7 | | |
|  | RNA/PEP1 | RNA/PEP2 | RNA/PEP1 | RNA/PEP2 | RNA/PEP3 | RNA/PEP1 | RNA/PEP2 | RNA/PEP3 |
| Correl WT | 0.02 | -0.22 | 0.94 | 0.96 | 0.99 | 0.45 | 0.34 | 0.59 |
| Correl NR | 0.59 | -0.81 | 0.91 | -0.36 | 0.93 | 0.53 | 0.40 | 0.89 |
| *P* WT  Power WT | *0.98*  0.026 | *0.75*  0.041 | *0.06*  0.412 | *0.04*  0.494 | *0.01*  0.754 | *0.55*  0.070 | *0.66*  0.054 | *0.41*  0.100 |
| *P* NR  Power NR | *0.41*  0.100 | *0.85*  0.202 | *0.09*  0.333 | *0.64*  0.057 | *0.07*  0.381 | *0.47*  0.085 | *0.60*  0.062 | *0.11*  0.295 |
