# Supplementary material for: Targeted Proteomics Allows Quantification of Ethylene Receptors and Reveals SlETR3 Accumulation in Never-Ripe Tomatoes
Source: Front Plant Sci. 2019 Aug 29;10:1054. doi: 10.3389/fpls.2019.01054 (PMC6727826; doi:10.3389/fpls.2019.01054)
Supplement: Supplementary file 1 [file DataSheet_1.zip › Figure S1 transitions for peptides.pptx]

## Slide 1
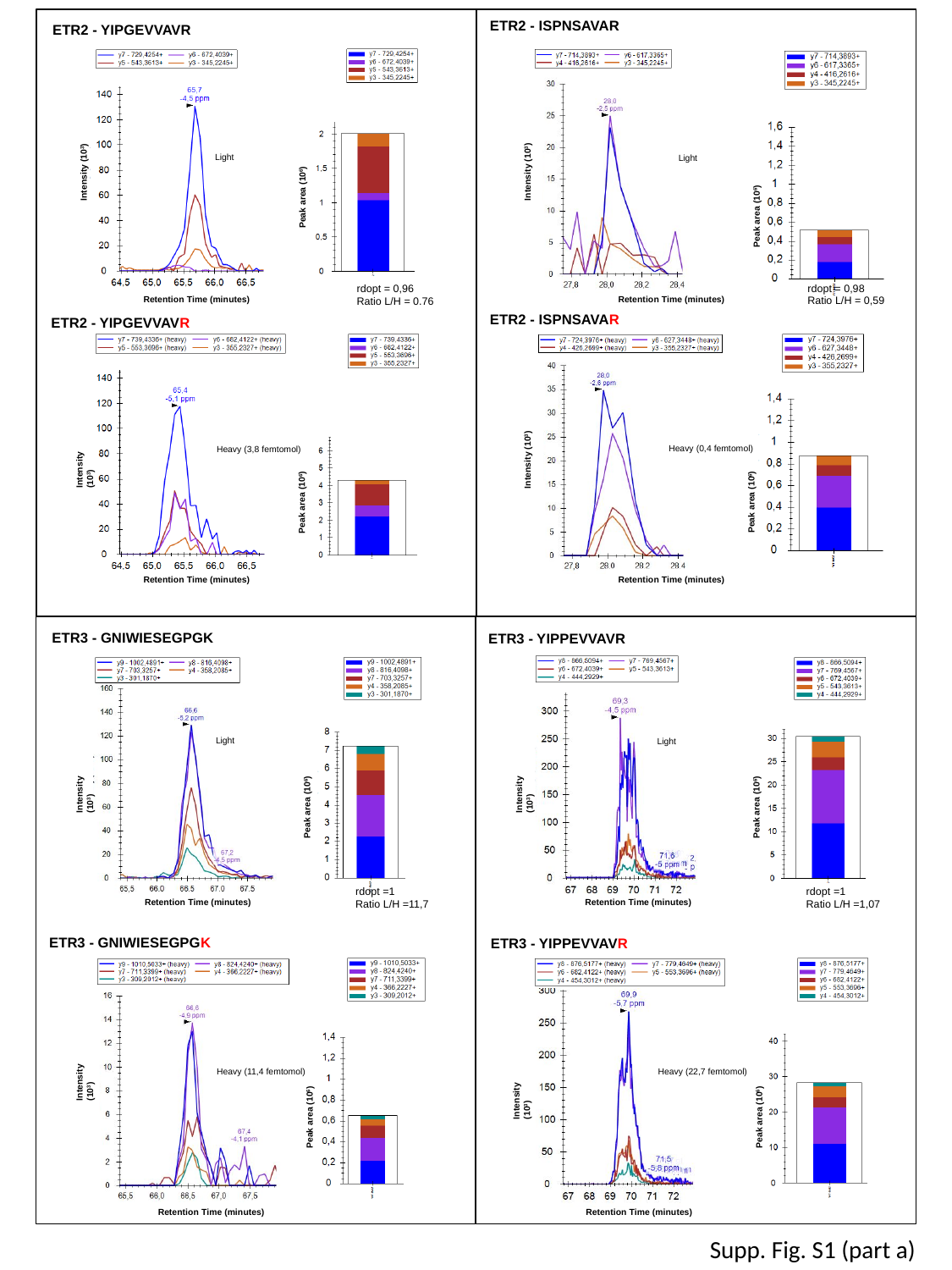

Light
Intensity (103)
Peak area (106)
rdopt = 0,96
Ratio L/H = 0.76
Retention Time (minutes)
ETR2 - YIPGEVVAVR
Heavy (3,8 femtomol)
Intensity (103)
Peak area (106)
Retention Time (minutes)
ETR2 - ISPNSAVAR
ETR2 - YIPGEVVAVR
Light
Intensity (103)
Peak area (106)
rdopt = 0,98
Ratio L/H = 0,59
Retention Time (minutes)
ETR2 - ISPNSAVAR
Heavy (0,4 femtomol)
Intensity (103)
Peak area (106)
Retention Time (minutes)
ETR3 - YIPPEVVAVR
Light
Intensity (103)
Peak area (106)
rdopt =1
Ratio L/H =1,07
Retention Time (minutes)
ETR3 - YIPPEVVAVR
Heavy (22,7 femtomol)
Intensity (103)
Peak area (106)
Retention Time (minutes)
ETR3 - GNIWIESEGPGK
Light
Intensity (103)
Peak area (106)
rdopt =1
Ratio L/H =11,7
Retention Time (minutes)
ETR3 - GNIWIESEGPGK
Intensity (103)
Heavy (11,4 femtomol)
Peak area (106)
Retention Time (minutes)
Supp. Fig. S1 (part a)

## Slide 2
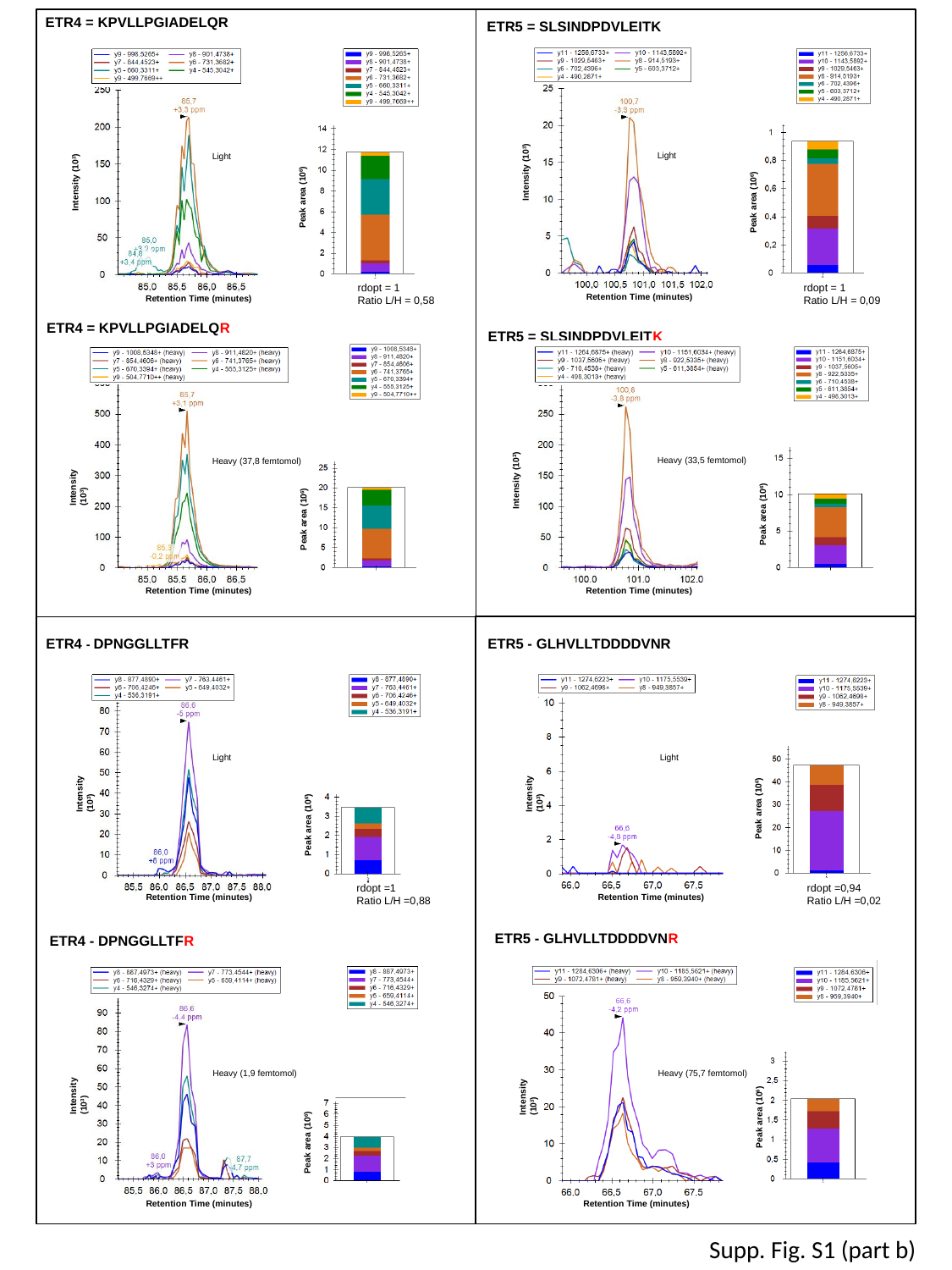

ETR4 = KPVLLPGIADELQR
Light
Intensity (103)
Peak area (106)
rdopt = 1
Ratio L/H = 0,58
Retention Time (minutes)
ETR4 = KPVLLPGIADELQR
Heavy (37,8 femtomol)
Intensity (103)
Peak area (106)
Retention Time (minutes)
ETR5 = SLSINDPDVLEITK
Light
Intensity (103)
Peak area (106)
rdopt = 1
Ratio L/H = 0,09
Retention Time (minutes)
ETR5 = SLSINDPDVLEITK
Heavy (33,5 femtomol)
Intensity (103)
Peak area (106)
Retention Time (minutes)
ETR5 - GLHVLLTDDDDVNR
Light
Intensity (103)
Peak area (106)
rdopt =0,94
Ratio L/H =0,02
Retention Time (minutes)
ETR5 - GLHVLLTDDDDVNR
Heavy (75,7 femtomol)
Intensity (103)
Peak area (106)
Retention Time (minutes)
ETR4 - DPNGGLLTFR
Light
Intensity (103)
Peak area (106)
rdopt =1
Ratio L/H =0,88
Retention Time (minutes)
ETR4 - DPNGGLLTFR
Heavy (1,9 femtomol)
Intensity (103)
Peak area (106)
Retention Time (minutes)
Supp. Fig. S1 (part b)

## Slide 3
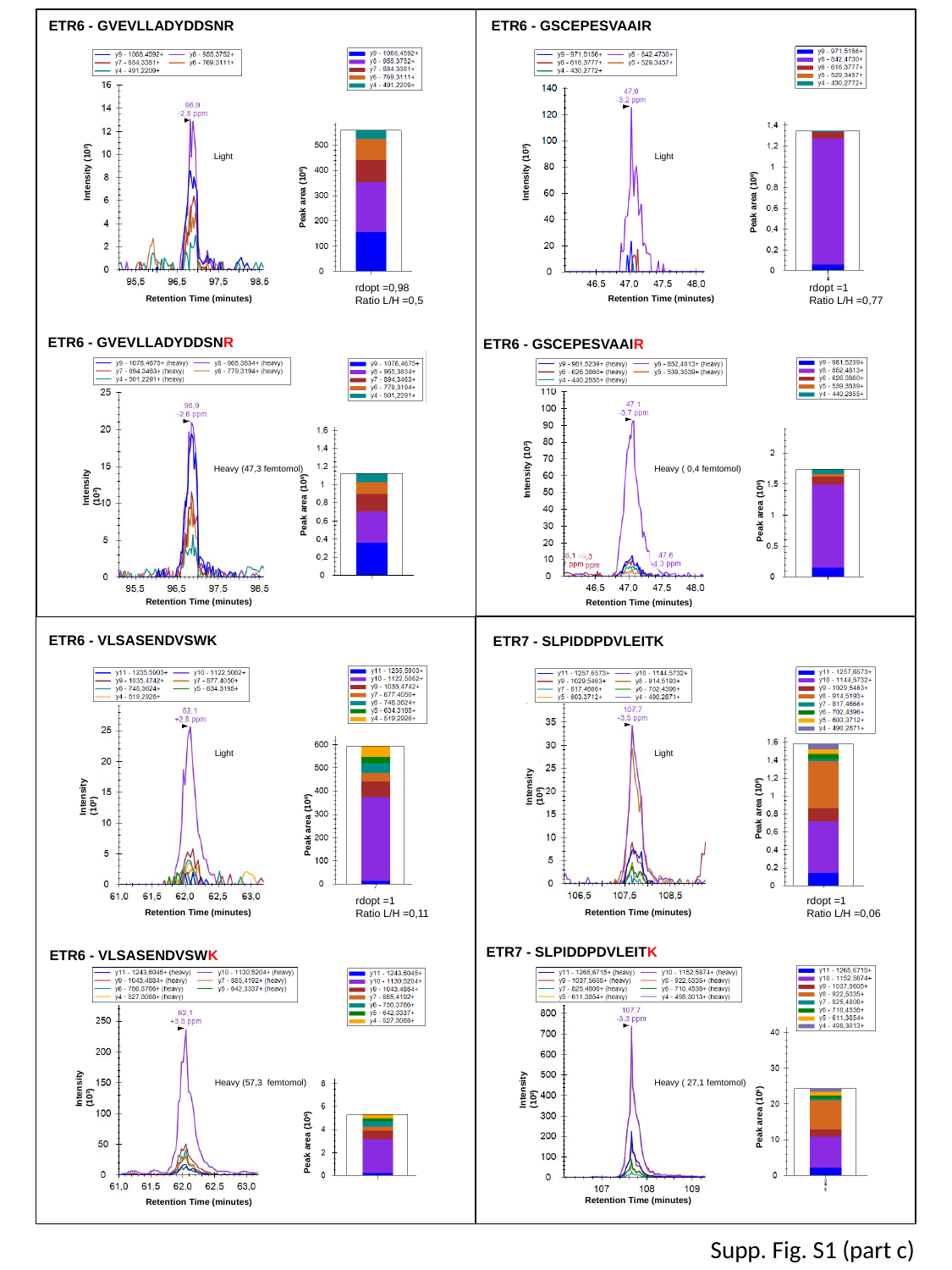

ETR6 - GSCEPESVAAIR
Light
Intensity (103)
Peak area (106)
rdopt =1
Ratio L/H =0,77
Retention Time (minutes)
ETR6 - GSCEPESVAAIR
Heavy ( 0,4 femtomol)
Intensity (103)
Peak area (106)
Retention Time (minutes)
ETR6 - GVEVLLADYDDSNR
Light
Intensity (103)
Peak area (106)
rdopt =0,98
Ratio L/H =0,5
Retention Time (minutes)
ETR6 - GVEVLLADYDDSNR
Heavy (47,3 femtomol)
Intensity (103)
Peak area (106)
Retention Time (minutes)
ETR7 - SLPIDDPDVLEITK
Light
Intensity (103)
Peak area (106)
rdopt =1
Ratio L/H =0,06
Retention Time (minutes)
ETR7 - SLPIDDPDVLEITK
Intensity (103)
Heavy ( 27,1 femtomol)
Peak area (106)
Retention Time (minutes)
ETR6 - VLSASENDVSWK
Light
Intensity (103)
Peak area (106)
rdopt =1
Ratio L/H =0,11
Retention Time (minutes)
ETR6 - VLSASENDVSWK
Intensity (103)
Heavy (57,3 femtomol)
Peak area (106)
Retention Time (minutes)
Supp. Fig. S1 (part c)

## Slide 4
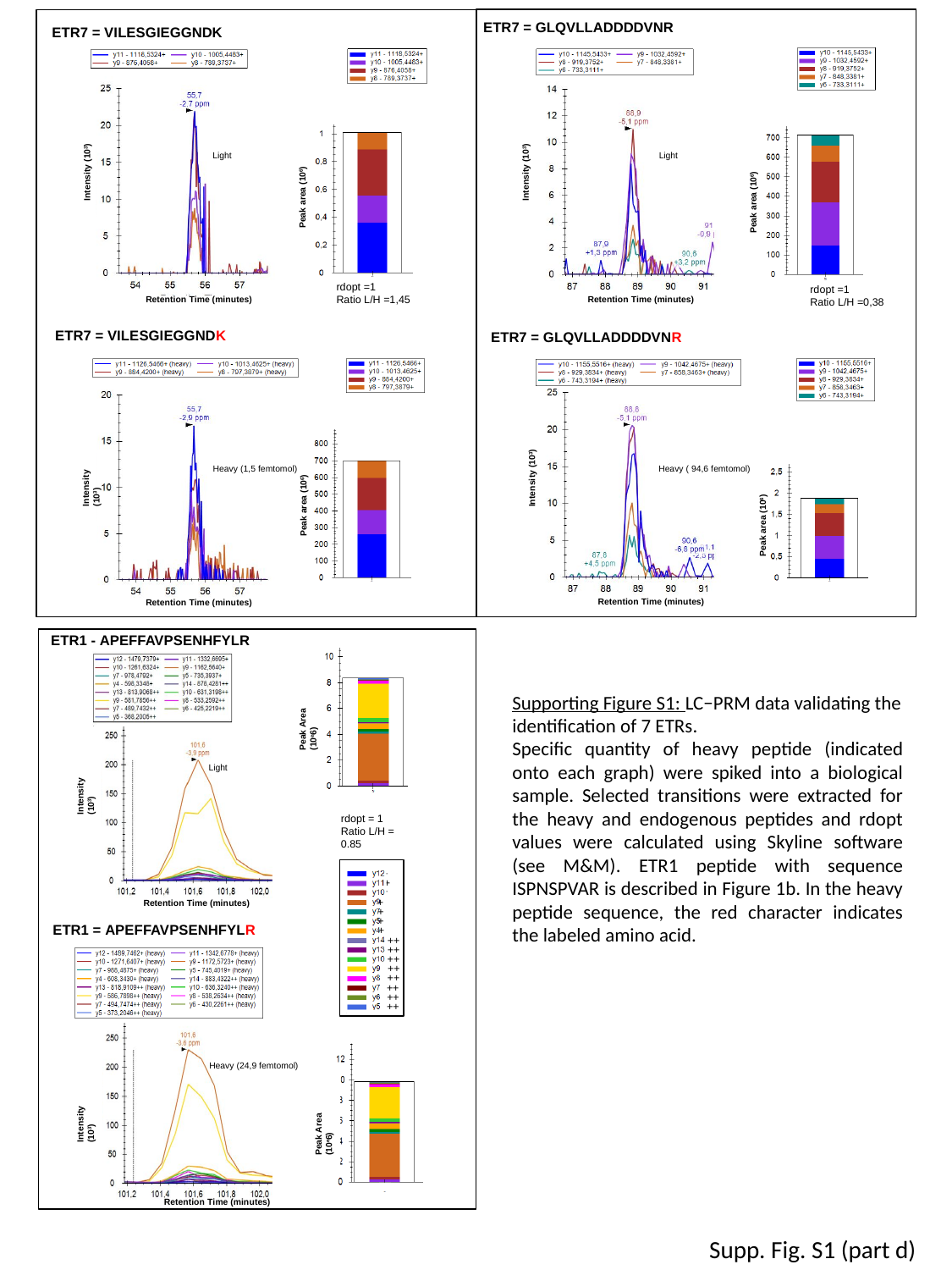

ETR7 = GLQVLLADDDDVNR
Light
Intensity (103)
Peak area (106)
rdopt =1
Ratio L/H =0,38
Retention Time (minutes)
ETR7 = GLQVLLADDDDVNR
Heavy ( 94,6 femtomol)
Intensity (103)
Peak area (106)
Retention Time (minutes)
ETR7 = VILESGIEGGNDK
Light
Intensity (103)
Peak area (106)
rdopt =1
Ratio L/H =1,45
Retention Time (minutes)
ETR7 = VILESGIEGGNDK
Heavy (1,5 femtomol)
Intensity (103)
Peak area (106)
Retention Time (minutes)
ETR1 - APEFFAVPSENHFYLR
Peak Area (10e6)
Light
Intensity (103)
rdopt = 1
Ratio L/H = 0.85
+
+
+
+
+
+
+
++
++
++
++
++
++
++
++
Retention Time (minutes)
ETR1 = APEFFAVPSENHFYLR
Heavy (24,9 femtomol)
Intensity (103)
Peak Area (10e6)
Retention Time (minutes)
Supporting Figure S1: LC−PRM data validating the identification of 7 ETRs.
Specific quantity of heavy peptide (indicated onto each graph) were spiked into a biological sample. Selected transitions were extracted for the heavy and endogenous peptides and rdopt values were calculated using Skyline software (see M&M). ETR1 peptide with sequence ISPNSPVAR is described in Figure 1b. In the heavy peptide sequence, the red character indicates the labeled amino acid.
Supp. Fig. S1 (part d)
