# Supplementary material for: Targeted Proteomics Allows Quantification of Ethylene Receptors and Reveals SlETR3 Accumulation in Never-Ripe Tomatoes
Source: Front Plant Sci. 2019 Aug 29;10:1054. doi: 10.3389/fpls.2019.01054 (PMC6727826; doi:10.3389/fpls.2019.01054)
Supplement: Supplementary file 1 [file DataSheet_1.zip › Figure S2 regressions for peptides.pptx]

## Slide 1
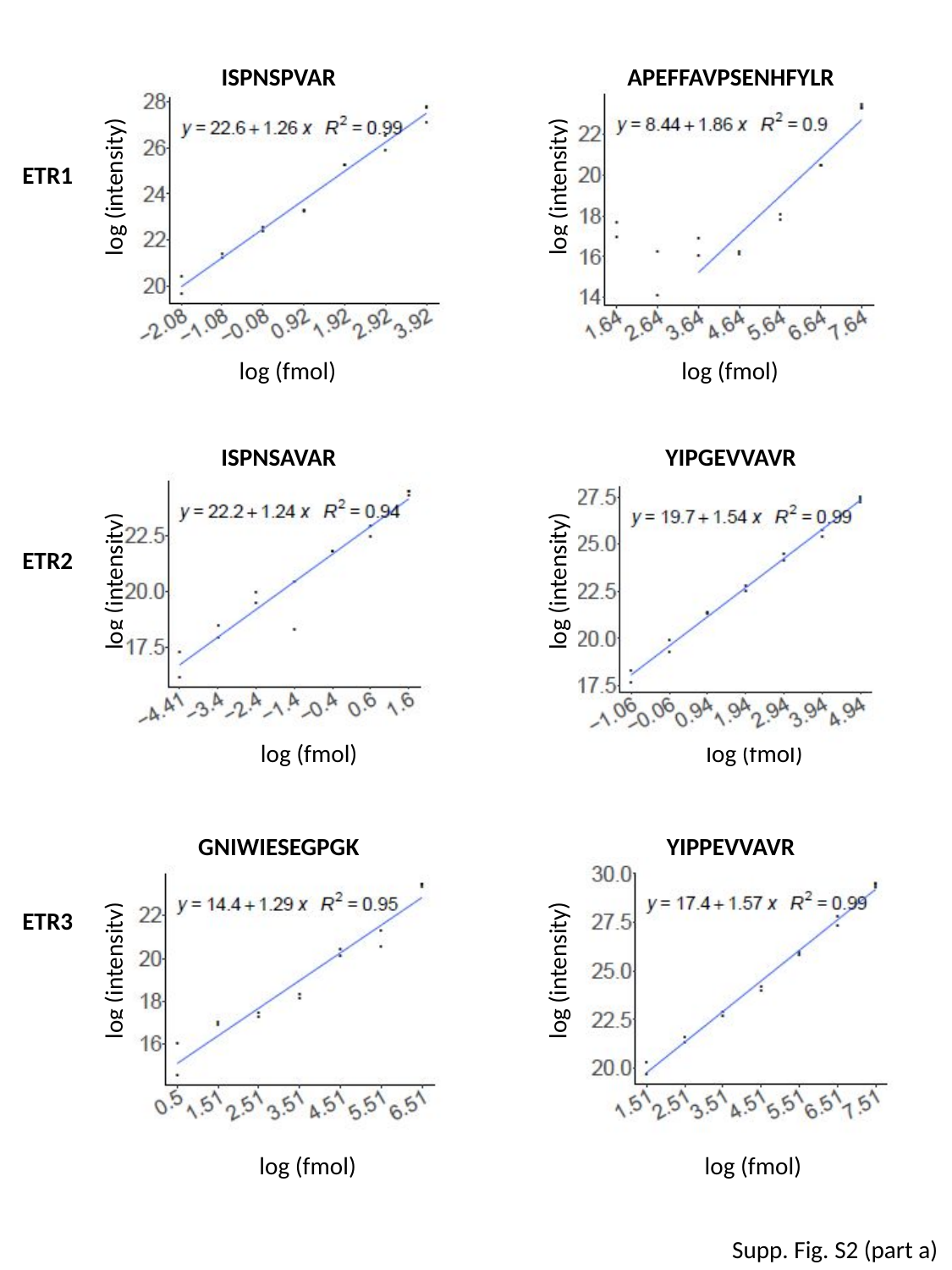

ISPNSPVAR
APEFFAVPSENHFYLR
ETR1
log (intensity)
log (intensity)
log (fmol)
log (fmol)
ISPNSAVAR
YIPGEVVAVR
ETR2
log (intensity)
log (intensity)
log (fmol)
log (fmol)
GNIWIESEGPGK
YIPPEVVAVR
ETR3
log (intensity)
log (intensity)
log (fmol)
log (fmol)
Supp. Fig. S2 (part a)

## Slide 2
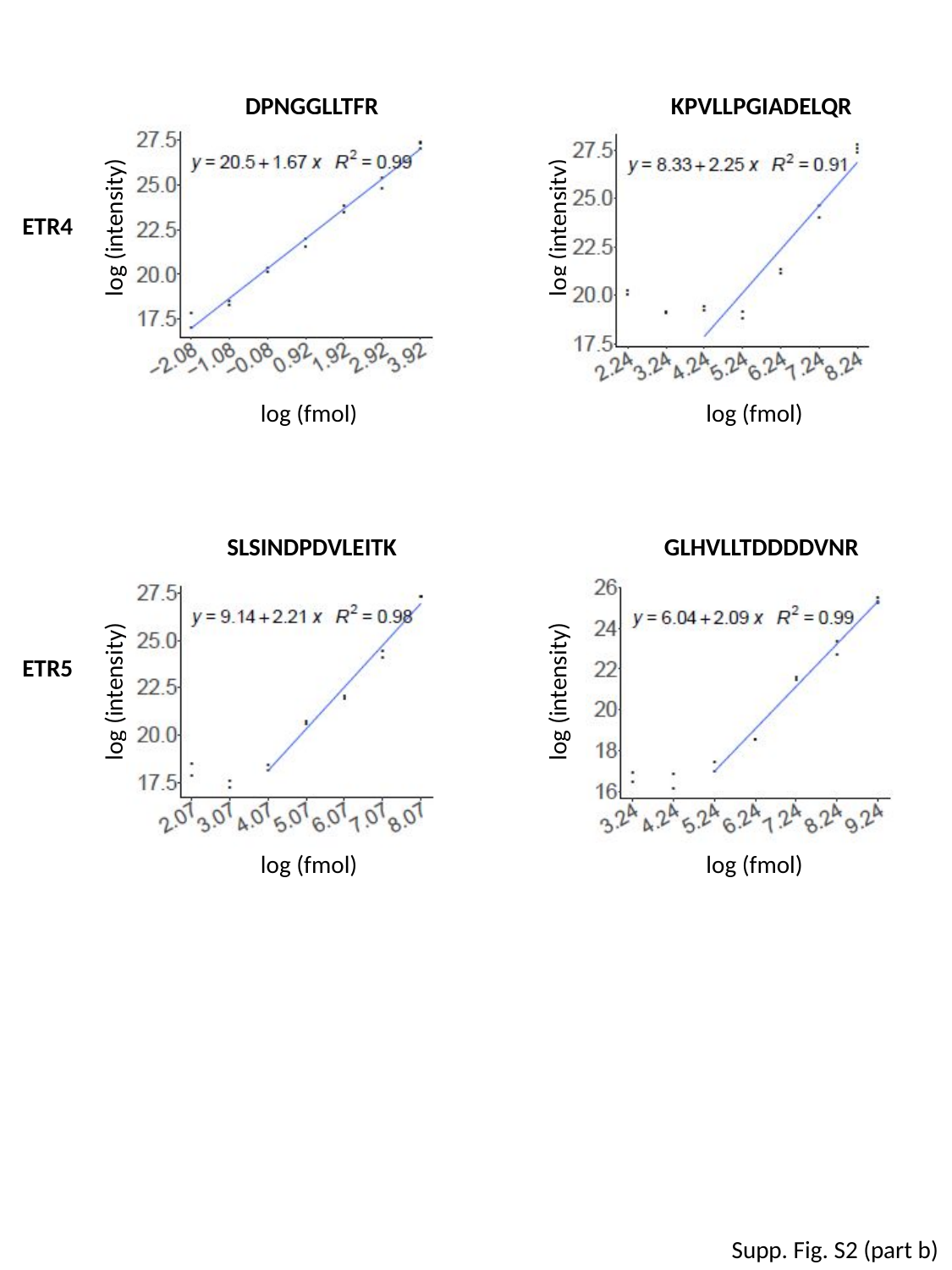

DPNGGLLTFR
KPVLLPGIADELQR
ETR4
log (intensity)
log (intensity)
log (fmol)
log (fmol)
SLSINDPDVLEITK
GLHVLLTDDDDVNR
ETR5
log (intensity)
log (intensity)
log (fmol)
log (fmol)
Supp. Fig. S2 (part b)

## Slide 3
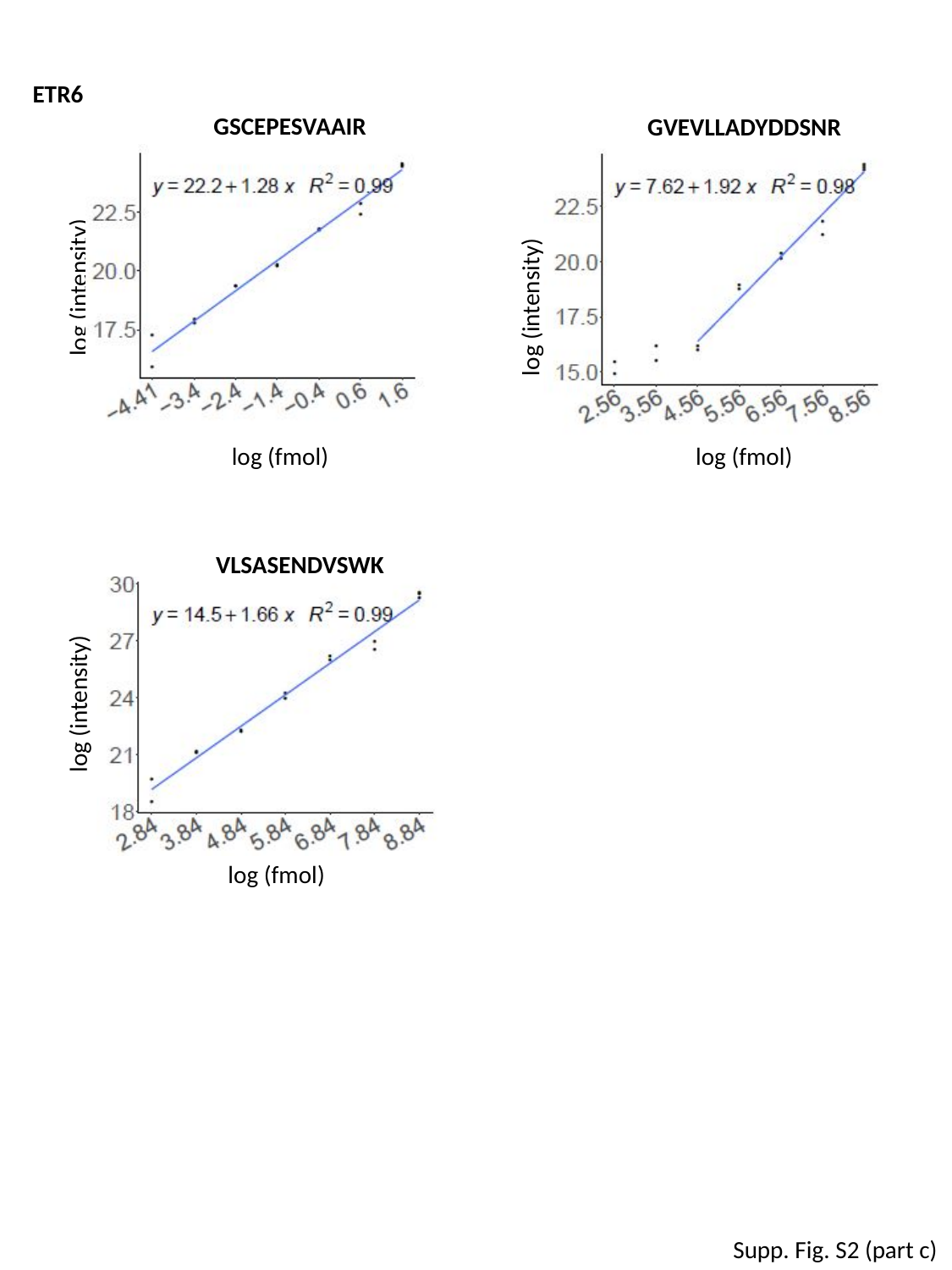

ETR6
GSCEPESVAAIR
GVEVLLADYDDSNR
log (intensity)
log (intensity)
log (fmol)
log (fmol)
VLSASENDVSWK
log (intensity)
log (fmol)
Supp. Fig. S2 (part c)

## Slide 4
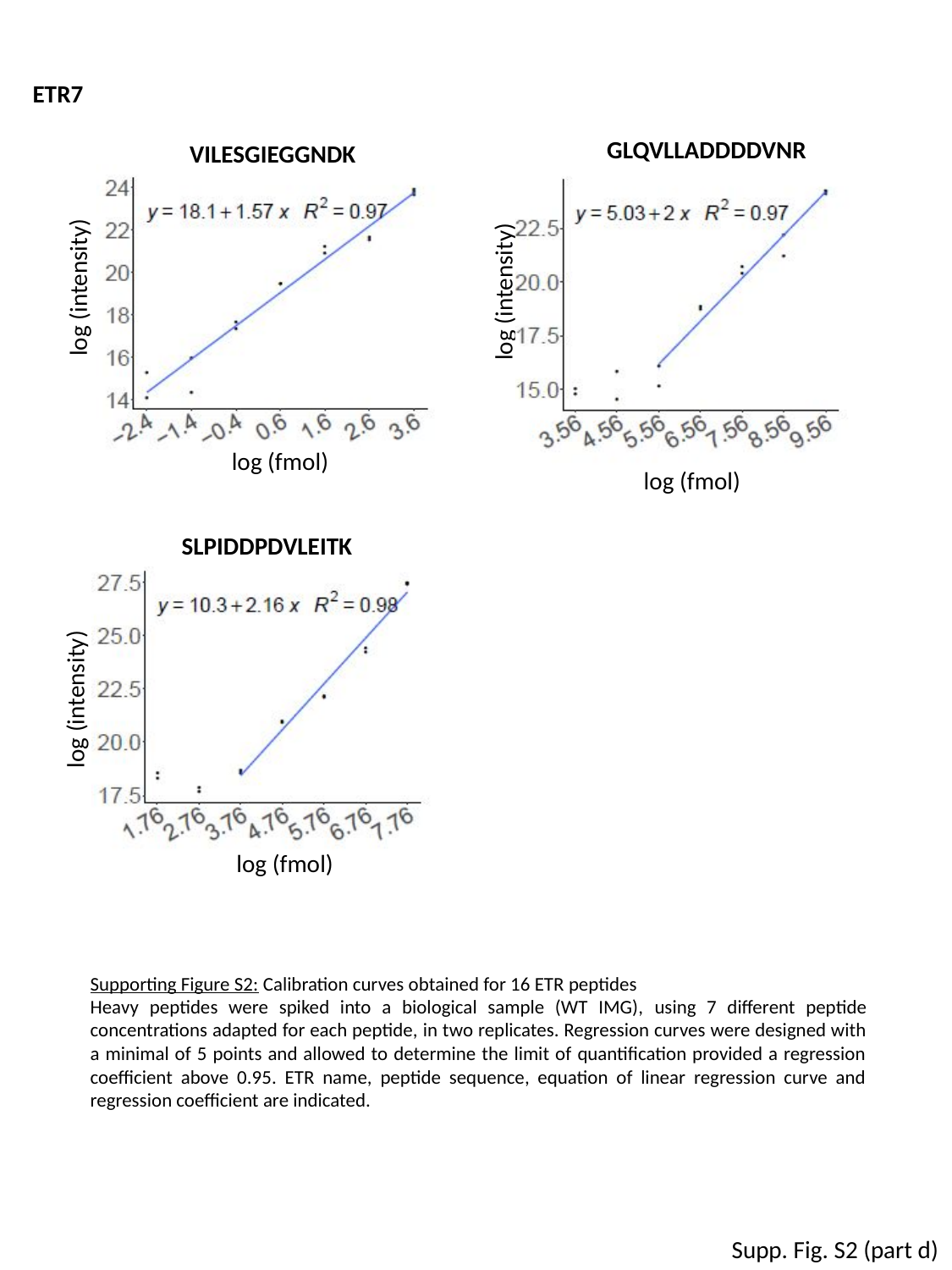

ETR7
GLQVLLADDDDVNR
VILESGIEGGNDK
log (intensity)
log (intensity)
log (fmol)
log (fmol)
log (fmol)
SLPIDDPDVLEITK
log (intensity)
log (fmol)
log (fmol)
Supporting Figure S2: Calibration curves obtained for 16 ETR peptides
Heavy peptides were spiked into a biological sample (WT IMG), using 7 different peptide concentrations adapted for each peptide, in two replicates. Regression curves were designed with a minimal of 5 points and allowed to determine the limit of quantification provided a regression coefficient above 0.95. ETR name, peptide sequence, equation of linear regression curve and regression coefficient are indicated.
Supp. Fig. S2 (part d)
