# Supplementary material for: Targeted Proteomics Allows Quantification of Ethylene Receptors and Reveals SlETR3 Accumulation in Never-Ripe Tomatoes
Source: Front Plant Sci. 2019 Aug 29;10:1054. doi: 10.3389/fpls.2019.01054 (PMC6727826; doi:10.3389/fpls.2019.01054)
Supplement: Supplementary file 1 [file DataSheet_1.zip › Methods S1.docx]

**Supporting Methods S1 a): Analytical details on the targeted nano LC-PRM**

-**Acquisition parameters:**

To target the 16 peptide sequences, following precursor ions including 16 light target masses

and 16 heavy target masses (-h) were included in the acquisition inclusion list. This list indicates *m/z*, charge state, polarity, scheduling start and end times, normalized collision energy (CE), amino acid sequence and precursor type (light form and heavy labeled).

For PRM acquisition, the same gradient was used as the one used to acquire HCD MS/MS reference spectra. This allowed deriving the retention times measured for the heavy labeled peptides spiked in a complex matrix to time-schedule the PRM assay. A 3 to 5 minute time window centered on the awaited retention time was set for each target. This setup leads to the following targets distribution over time.


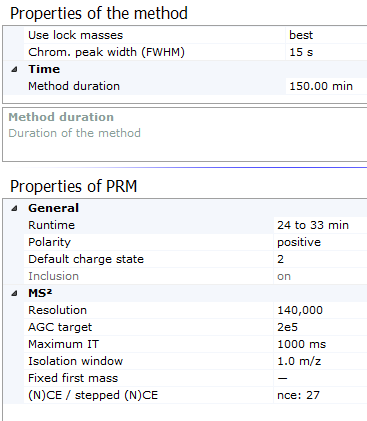

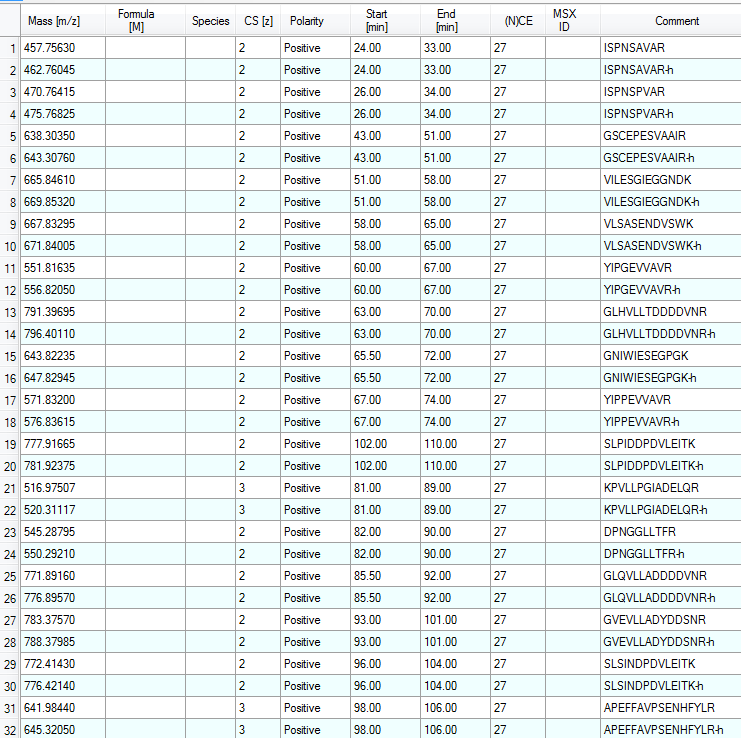


**-Analysis parameters:**

For spectral library building, MS/MS data were interrogated using a local Mascot server (version 2.4.1, Matrix Science, London, U.K.) against a database containing the 7 ETRs proteins. The following parameters were used: trypsin as enzyme, 10 ppm in MS and 0.02 Da in MS/MS, carbamidomethylation (C) as fixed modification and label : ^13^C6,^15^N2 (K) and ^13^C6,^15^N4 (R) as variable modifications. In Skyline, precursor and product ion masses were selected in the spectral library with the following settings: monoisotopic masses, precursor charges +2 and +3, product ion charges +1, +2, from ion 2 to last ion.

**Supporting Methods S1 b): Large scale quantitative label-free proteomics**

Gel band treatments and trypsin digestion were performed mainly as described in ([Geiger *et al.* 2015](#_ENREF_2)). Briefly, proteins in the gel slices were reduced, alkylated, and digested overnight at 37 °C with modified trypsin at a 1:100 enzyme/ protein ratio (Promega, Madison, WI). Peptides were extracted twice by addition of 200 µL of 80 % acetonitrile (ACN) and 2% formic acid (FA), and then dried in a vacuum centrifuge. Peptides were then resuspended in 20 µl FA 2% before LC-MS/MS analysis. The LC-MS/MS experiments were performed in Data Dependent Acquisition (DDA) mode, using an UltiMate™ NCS-3500RS Ultra High Performance Liquid Chromatography (Thermo Fisher Scientific Inc, Waltham, MA, USA, C18, 2 μm particle size, 100 Å pore size, 75 μm i.d. x 50 cm length) system interfaced online with a nano easy ion source and a Q Exactive Plus Orbitrap mass spectrometer (ThermoFisher Scientific Inc, Waltham, MA, USA). Peptides were first loaded onto a pre-column (Thermo Scientific PepMap 100 C18, 5 μm particle size, 100 Å pore size, 300 μm i.d. x 5 mm length) from the Ultimate 3000 autosampler with 0.05% TFA for 3 min at a flow rate of 10 μL/min. Then, the column valve was switched to allow elution of peptides from the pre-column onto the analytical column. Loading buffer (solvent A) was 0.1% FA and elution buffer (solvent B) was 80% ACN + 0.1% FA. The 3 step gradient employed was 4-25% of solvent B for 103 min, then 25-40% of solvent B up to 123 min, and 40-90% of solvent B from 123 to 125 min, at a flow rate of 300 nL/min. The total chromatographic run time was 150 min including a high organic wash step and re-equilibration step. Peptides were transferred to the gaseous phase with positive ion electrospray ionization at 1.7 kV. In such a DDA mode the top 10 precursors were acquired between 375 and 1500 m/z with an isolation windows of 2 *m/z*, a dynamic exclusion of 40 s, a normalized collision energy of 27 and resolutions of 70,000 and 17,500 for MS and MS2, respectively.

The *.raw* files were analyzed with MaxQuant version 1.5.5.1 using default settings. The minimal peptide length was set to 6. The criteria “Trypsin/P” (which means C-terminus peptides of “K/R” unless followed by “P”: “K/R” followed by “P” cannot be a cleavage site) was chosen as digestion enzyme. Carbamidomethylation of cysteine was selected as a fixed modification and oxidation of methionine, deamidation of asparagine and glutamine, N-terminal-pyroglutamylation of glutamine and glutamate and acetylation (protein N terminus) as variable modifications. Up to two missed cleavages were allowed. The mass tolerance for the precursor was 20 and 4.5 ppm for the first and the main searches respectively, and for the fragment ions was 20 ppm. The files were searched against Solanum lycopersicum \ITAG3.2 database (<ftp://ftp.solgenomics.net/tomato_genome/annotation/ITAG3.2_release/>). Identified proteins were filtered according to the following criteria: at least two different trypsin peptides with at least one unique peptide, an E value below 0.01 and a protein *E* value smaller than 0.01 were required. Using the above criteria, the rate of false peptide sequence assignment and false protein identification were lower than 1%.

Peptide ion intensity values derived from MaxQuant were subjected for label-free quantitation. Unique and razor peptides were considered ([Cox & Mann, 2008](#_ENREF_1)). Statistical analyses were carried out using R software. ANOVA test controlled by Benjamini–Hochberg FDR threshold of 0.05 was applied to identify the significant differences in the protein abundance. Hits were retained if they were quantified in at least two of the three replicates in at least one experiment. The data are available in Supp. Table S2b.

*Additional references:*

**Cox J, Mann M. 2008.** MaxQuant enables high peptide identification rates, individualized p.p.b.-range mass accuracies and proteome-wide protein quantification. *Nature biotechnology* **26**: 1367–1372.

**Geiger A, Hamidou Soumana I, Tchicaya B, Rofidal V, Decourcelle M, Santoni, V, Hem S. 2015.** Differential expression of midgut proteins in Trypanosoma brucei gambiense-stimulated vs. non-stimulated Glossina palpalis gambiensis flies. *Frontiers in Microbiology* **6**: 444.
