# Supplementary material for: Targeted Proteomics Allows Quantification of Ethylene Receptors and Reveals SlETR3 Accumulation in Never-Ripe Tomatoes
Source: Front Plant Sci. 2019 Aug 29;10:1054. doi: 10.3389/fpls.2019.01054 (PMC6727826; doi:10.3389/fpls.2019.01054)
Supplement: Supplementary file 1 [file DataSheet_1.zip › Table S1 specif peptides and qPCR primers.docx]

**Supporting Table S1**: **a)** List of labeled ETR peptides used for PRM analysis

| Protein | Peptide sequence | *z* | Average RT (min) | *m/z* | rdopt |
| --- | --- | --- | --- | --- | --- |
| SlETR1 (Solyc12g011330) | ISPNSPVAR | 2 | 30 | 475.768 | 1 |
|  | APEFFAVPSENHFYLR | 3 | 102 | 645.321 | 1 |
| SlETR2 (Solyc07g056580) | ISPNSAVAR | 2 | 28.5 | 462.761 | 0.99 |
|  | YIPGEVVAVR | 2 | 63.5 | 556.821 | 0.96 |
| SlETR3 (Solyc09g075440) | GNIWIESEGPGK | 2 | 69 | 647.830 | 1 |
|  | YIPPEVVAVR | 2 | 70.5 | 576.836 | 1 |
| SlETR4 (Solyc06g053710) | DPNGGLLTFR | 2 | 86 | 550.292 | 1 |
|  | KPVLLPGIADELQR | 3 | 85 | 520.311 | 1 |
| SlETR5 (Solyc11g006180) | SLSINDPDVLEITK | 2 | 100 | 776.421 | 1 |
|  | GLHVLLTDDDDVNR* | 2 | 66.5 | 796.401 | 0.94 |
| SlETR6 (Solyc09g089610) | GSCEPESVAAIR | 2 | 47 | 643.308 | 1 |
|  | VLSASENDVSWK | 2 | 61.5 | 671.840 | 1 |
|  | GVEVLLADYDDSNR | 2 | 197 | 788.380 | 0.98 |
| SlETR7 (Solyc05g055070) | VILESGIEGGNDK | 2 | 54.5 | 665,846 | 1 |
|  | GLQVLLADDDDVNR | 2 | 89 | 771,891 | 1 |
|  | SLPIDDPDVLEITK | 2 | 106 | 781.924 | 1 |

Labeled ETR were checked according to their charge (*z*), their average retention time (RT) and *m/z*. The traces for the heavy and endogenous peptides were extracted (Fig. 2, Supp. Fig. S1) and rdopt-product values (last column) were calculated using Skyline software. * indicates not reliable peptides because of rdopt < 0.95.

**Supporting Table S1**: **b)** Peptide number used for the calculation of Pearson correlation coefficients (in Fig. 2)

| **Receptors** | **Peptide 1** | **Peptide 2** | **Peptide 3** |
| --- | --- | --- | --- |
| **SlETR1** | ISPNSPVAR | APEFFAVPSENHFYLR |  |
| **SlETR2** | ISPNSAVAR | YIPGEVVAVR |  |
| **SlETR3** | GNIWIESEGPGK | YIPPEVVAVR |  |
| **SlETR4** | DPNGGLLTFR | KPVLLPGIADELQR |  |
| **SlETR5** | SLSINDPDVLEITK | GLHVLLTDDDDVNR |  |
| **SlETR6** | GSCEPESVAAIR | VLSASENDVSWK | GVEVLLADYDDSNR |
| **SlETR7** | VILESGIEGGNDK | GLQVLLADDDDVNR | SLPIDDPDVLEITK |

**Supporting Table S1**: **c)** Primer sequences for qPCR

| **Primer name** | **Sequence (5’→3’)** |
| --- | --- |
| qETR1-F | GCCTTTTATCTTCCATCGTGGA |
| qETR1-R | GATACTTCATTAGCAAGTCGTCAGCA |
| qETR2-F | TGGCATTCCTGGTCGCTTA |
| qETR2-R | TCTGCATGTGATTTGCAGGC |
| qETR3-F | GCTTTGGCTCTGGATTTACCTATTC |
| qETR3-R | TTCCCGCCACGTTTAAGAGA |
| qETR4-R | CCACAACCCTGACTATCTCAATTTC |
| qETR4-F | GCCATACTGGTTTTGGTTCTACCTA |
| qETR5-F | TGTTCAGATGATGCAGGGAAAT |
| qETR5-R | ATGAGTGTCATCCCCTGCG |
| qETR6-F | AAAAGCCGGTGATCTCGGTA |
| qETR6-R | AAACTAGAACAGGAAACGAAGTAGATGA |
| qETR7-F | CGCTTTTCCACGGATGTTCC |
| qETR7-R | ATCATTCCCGCCCTCGATTC |
| qActin-F | TGTCCCTATTTACGAGGGTTATGC |
| qActin-R | CAGTTAAATCACGACCAGCAAGAT |
| qEF1α-F | GAAGATGATTCCCACCAAGC |
| qEF1α-R | TGACACCAACAGCCACAGTT |
| qGAPDH-F | CTGCTCACTTGAAGGGTGGT |
| qGAPDH-R | GACAATGTCCAGCTCTGGCT |
